# Neonatal Ventricular Reservoir Implantation for Hydrocephalus Management in Chiari III Malformations: A Case Report

**DOI:** 10.7759/cureus.55896

**Published:** 2024-03-10

**Authors:** Risa Ito, Yutaro Fuse, Keishi Ito, Hisashi Hatano, Ryuta Saito

**Affiliations:** 1 Department of Neurosurgery, Japanese Red Cross Aichi Medical Center Nagoya Daiichi Hospital, Nagoya, JPN; 2 Department of Neurosurgery, Nagoya University Graduate School of Medicine, Nagoya, JPN; 3 Academia-Industry Collaboration Platform for Cultivating Medical AI Leaders, Nagoya University, Nagoya, JPN

**Keywords:** chiari malformations, pediatric hydrocephalus, reservoir, ventriculoperitoneal (vp) shunt, chiari iii malformation

## Abstract

Chiari III malformation, a rare and severe subtype of Chiari malformations, is frequently associated with hydrocephalus. The conventional treatment approaches for hydrocephalus in Chiari III malformations have mainly focused on ventriculoperitoneal (VP) shunting, but optimal methods and timing remain uncertain. We report a case of a newborn girl with Chiari III malformation who underwent surgical closure of an occipitocervical encephalocele and ventricular reservoir implantation on her third day of life. This procedure successfully managed her hydrocephalus without significant short-term complications. Three months post-surgery, she developed secondary external hydrocephalus, which was managed through subdural-peritoneal shunting, allowing her to thrive until at least five years of age. This case demonstrates the potential of ventricular reservoir implantation in treating hydrocephalus associated with Chiari III malformation and brings to light secondary external hydrocephalus, subsequently managed by VP shunting.

## Introduction

Chiari III malformation, a rare and severe developmental anomaly, is characterized by the downward displacement of cerebellar tonsils and the presence of encephaloceles, often accompanied by hydrocephalus [1,2]. The primary surgical goals in treating patients with Chiari III malformation include extensive resection and repair of the encephalocele, preservation of neurological function, normalization of cerebrospinal fluid (CSF) circulation, and prevention of future tethering of the spinal cord and brainstem [2,3]. Current interventions for hydrocephalus comprise ventriculoperitoneal (VP) shunting and external drainage [2,4-6]. However, the optimal timing for these interventions remains unclear, and reports on alternative methods for managing CSF flow and their long-term postoperative outcomes in Chiari III malformation are limited.

In this report, we describe a case of Chiari III malformation where surgical closure of an encephalocele and implantation of a ventricular reservoir were performed on the third day after birth. This approach resulted in a notable improvement in hydrocephalus symptoms. Three months later, subdural-peritoneal shunting was performed to address external hydrocephalus, enabling the patient to thrive and continue to do so for over five years, despite experiencing delayed psychomotor development.

## Case presentation

**Figure 1:**
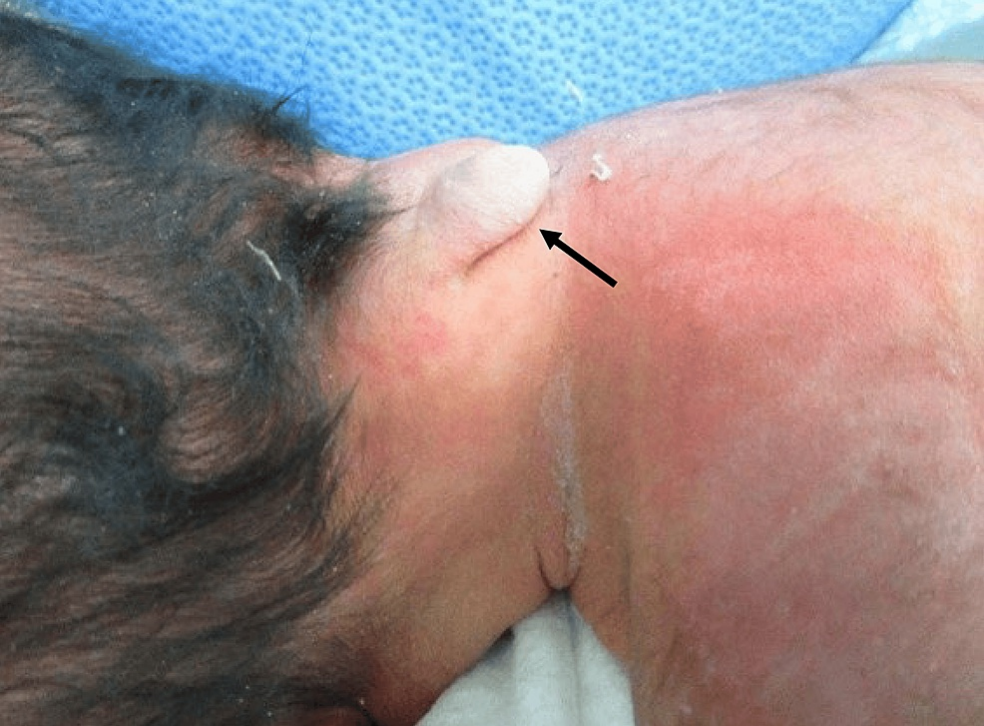
The photograph displays a cystic occipitocervical mass The black arrow points to a mass measuring 20 × 20 × 20 mm.

The patient is a newborn female who was delivered via cesarean section at 36 weeks of gestation due to concerns regarding the risk of CSF leakage and trauma to the central nervous system. Hydrocephalus and an occipitocervical encephalocele were identified at 25 weeks gestation through maternal transabdominal ultrasonography. The biparietal diameter measured 2.3 standard deviations at that time. Her Vietnamese mother and Japanese father had no notable family medical history. At birth, she scored 8/9 on the Apgar scale, weighed 3549 grams, and had a head circumference of 44.2 cm. She exhibited a 20 mm occipitocervical mass covered in normal skin (Figure 1). She displayed no neurological or respiratory impairments.

**Figure 2:**
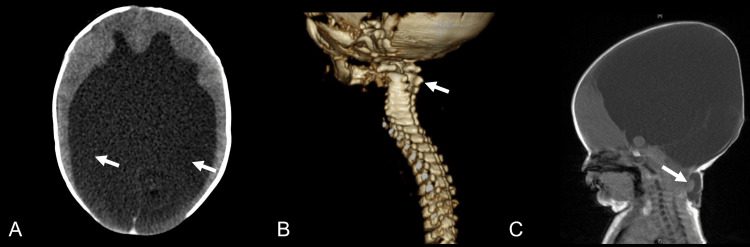
Imaging study findings at birth A) CT scan showing hydrocephalus (white arrows); B) CT scan revealing a defect (white arrow) in the posterior arch from C1 to C3; C) Sagittal MRI illustrating a high cervical herniation (white arrow) into the encephalocele CT, computed tomography; MRI, magnetic resonance imaging

CT scans revealed substantially enlarged supratentorial ventricles, a normally formed occipital bone, and a defect in the posterior arch from C1 to C3 (Figures 2A, 2B). MRI findings included a high cervical herniation into the encephalocele, suggesting the presence of cerebellar or other brain tissues (Figure 2C), thus confirming a diagnosis of Chiari III malformation with concurrent hydrocephalus.

**Figure 3:**
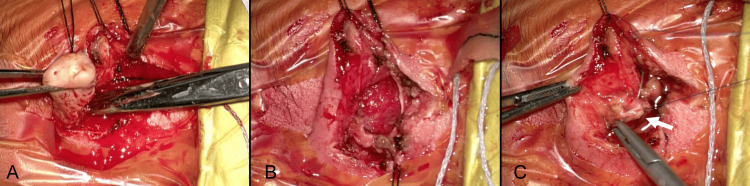
Intraoperative images from the primary surgery A) Skin incision made around the encephalocele; B) Exposure of the encephalocele contents; C) Closure of the dura mater (white arrow)

**Figure 4:**
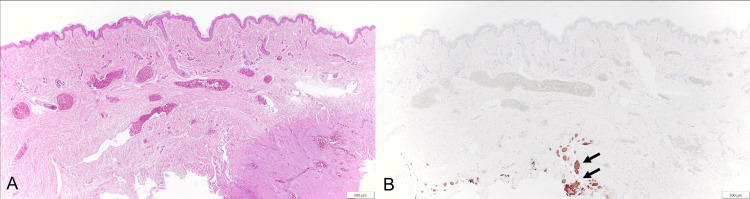
Sections of the excised specimen A) Hematoxylin and eosin staining; B) GFAP staining (the black arrows indicate GFAP-positive cells) GFAP, glial fibrillary acidic protein

At three days old, the patient underwent surgical treatment. To restore normal CSF circulation and encephalocele closure at this early day of life, a ventricular reservoir placement procedure was utilized. The procedure began with an arcuate incision over the anterior fontanel in a supine position to expose the right parietal bone. The opening was enlarged using bipolar coagulation. When the dura mater was incised, cerebrospinal fluid released under high pressure caused the anterior fontanel to collapse. An Ommaya Neonate reservoir, measuring 4 cm in length, was then inserted into the ventricle, and the reservoir itself was positioned over the parietal bone. The galea and scalp were sutured continuously. The patient was then repositioned prone for encephalocele closure, which involved a spindle-shaped incision around the encephalocele, dissection of subcutaneous tissue (Figure 3A), exposure of encephalocele contents (Figure 3B), and detaching the vascular tissue tethering the sac, followed by dural and skin closure (Figure 3C). Pathological examination of the excised skin tissue showed a normal epidermis with glial fibrillary acidic protein (GFAP)-positive central nervous tissue (Figure 4).

**Figure 5:**
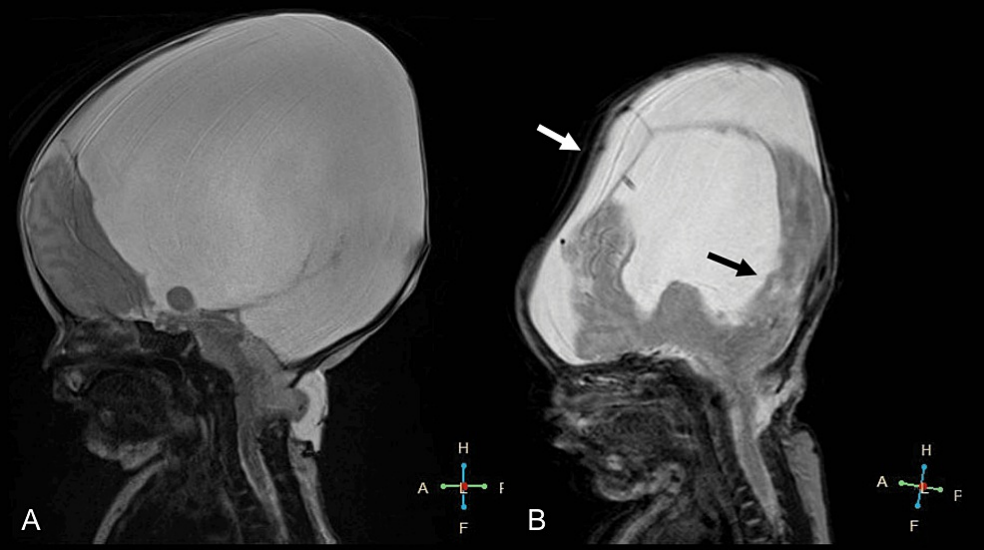
Comparison of pre- and post-procedural MRI T2 sagittal images of the head A) Pre-procedural MRI; B) Post-procedural MRI, the black arrow highlights the recovery of brain volume and the white arrow points to the improvement in hydrocephalus MRI, magnetic resonance imaging

Postoperatively, by day 11, her head circumference had reduced to 34.3 cm. As compared to the preoperative MRI (Figure 5A), the postoperative MRI (Figure 5B) showed an improvement in hydrocephalus findings, with an increase in brain volume. The cerebellar herniation had resolved at the surgical site following the repair of the meningeal mass, with the spinal fluid cavity at the craniocervical junction showing signs of normalization. The patient experienced a favorable postoperative recovery and was discharged on day 14.

**Figure 6:**
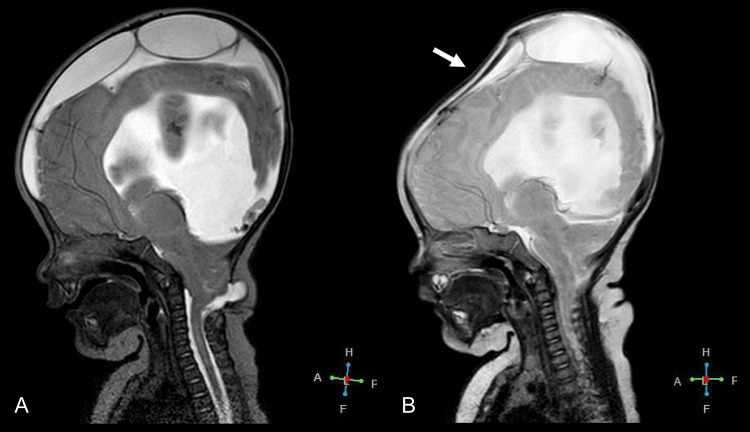
Comparison of MRI T2 sagittal images of the head before and after the subdural-peritoneal shunting procedure A) MRI before the subdural-peritoneal shunting; B) MRI after the subdural-peritoneal shunting, the white arrow points to the shrinkage of the external hydrocephalus MRI, magnetic resonance imaging

At 93 days old, she was readmitted due to vomiting and increased head circumference. She weighed 7.14 kg and was 58.1 cm tall. Her developmental milestones included feeding approximately 10 times a day, making eye contact, and smiling, but she was unable to hold her neck steady or turn over. Upon chest auscultation, no heart murmur was detected, and her abdomen was observed to be flat and soft. MRI revealed external hydrocephalus (Figure 6A). A left subdural-peritoneal shunt procedure was performed, which immediately relieved her symptoms. An MRI conducted on the sixth day after the operation showed a bilateral reduction in subdural space and a recovery in brain volume (Figure 6B).

**Figure 7:**
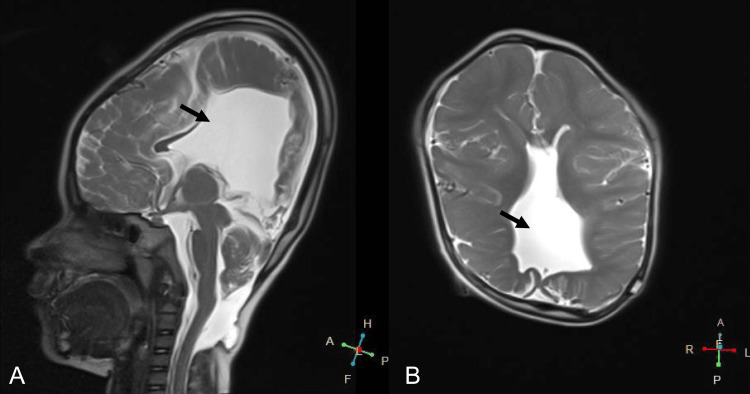
MRI T2 imaging at the age of four A) Sagittal view, the black arrow highlights the ventricle remaining reduced in size; B) Axial view, the black arrow highlights the ventricle remaining reduced in size MRI, magnetic resonance imaging

At the age of three years and five months, she underwent shunt reconstruction due to shunt dysfunction, performed through a right posterior horn puncture. An MRI at four years old confirmed that the ventricles remained reduced in size (Figure 7). By five years and six months, despite delayed psychomotor development, she could orally ingest food, turn over, raise her upper limbs, and grasp objects.

Written informed consent to participate in this case report was obtained from the person with parental authority.

## Discussion

In this study, we encountered a case of Chiari III malformation where a significant improvement in hydrocephalus was achieved following surgical closure of an encephalocele and implantation of a ventricular reservoir on the third-day post-birth. The patient later underwent shunting to address external hydrocephalus, enabling her to thrive and continue to do so for over five years.

Chiari malformations encompass a group of disorders characterized by the descent of the cerebellum and brainstem through the foramen magnum into the spinal canal. Among these, type III malformations stand out as a particularly rare and severe form, first identified in 1891 [7]. Chiari Type III malformation is defined by a defect in the occipital bone or upper cervical vertebrae, leading to the extrusion of intracranial contents as a high cervical/occipital encephalocele [8]. In this case, a 2 cm mass was noted in the upper cervical region, with the excised specimen revealing GFAP-positive CNS cells, aligning with encephalocele characteristics. The severity of symptoms often correlates with the amount of brain tissue present within the encephalocele [9]. The relatively minor size of the mass in this instance compared to previous reports [8,10] might have contributed to the preservation of brainstem-controlled swallowing functions. Furthermore, while encephalocele contents can sometimes undergo necrosis [11], there was no evidence of such a process in this case.

The novelty of this report lies in the implementation of a ventricular reservoir as a treatment for hydrocephalus in Chiari III malformations. Typically, Chiari III malformation treatment involves surgical repair of the encephalocele and management of hydrocephalus if present. The conventional methods for these cases have primarily been VP shunting and external drainage [2,6]. However, VP shunting is associated with issues such as infection and excessive fluid outflow [12,13]. External drainage is typically followed by shunting, and reports of long-term implantation are scarce. In contrast, the placement of a ventricular reservoir is recognized for its advantages over VP shunting in managing hydrocephalus immediately after birth in various other conditions [14]. In this case, since hydrocephalus was prenatally diagnosed, we deemed it crucial to address the condition within the first few days of life. A ventricular reservoir is reported to effectively reduce ventricular size and intracranial pressure [15], and we believe that its placement played a pivotal role in the rapid improvement of hydrocephalus in this case as well.

The patient underwent encephalocele closure and ventricular reservoir implantation three days after birth. Immediate surgical intervention is typically considered in cases with signs of respiratory distress or thinning skin over the encephalocele. Our patient exhibited neither, displaying normal skin and no respiratory issues, thus not requiring emergency surgery [2]. The diagnosis of Chiari malformation is typically established through fetal MRI or ultrasound [16]. In our patient, the diagnosis had already been confirmed via ultrasound during pregnancy. Consequently, we elected to operate three days after birth. Using a ventricular reservoir allowed for simultaneous encephalocele closure and restoration of normal CSF circulation at this early day of life. This integrated approach enabled us to achieve the surgical goals outlined in prior literature [3], including extensive encephalocele resection and repair, neurological function preservation, normal CSF circulation restoration, and future spinal cord and brainstem tethering prevention, all with minimal short-term complications. Indeed, the patient was discharged within two weeks post-surgery.

Three months after the initial surgery, the patient developed secondary external hydrocephalus. This condition is caused by the formation of a communication pathway between the subdural and subarachnoid spaces, often due to an opening in the arachnoid membrane [17]. In this case, the onset of external hydrocephalus could likely be ascribed to an arachnoid fistula that may have been inadvertently created during the initial surgical procedure. This arachnoid fistula might have interfered with the normal CSF flow during the healing process. Furthermore, any inherent disproportion between the intracranial space and cerebral tissue, which could be congenital, might have been exacerbated by the loss of cerebrospinal fluid, contributing further to the development of external hydrocephalus.

The patient underwent shunt surgery to treat secondary external hydrocephalus. While we initially intended to consider shunting as a standby measure around six months of age, symptomatic external hydrocephalus necessitated its earlier performance. In Chiari I malformations, external hydrocephalus post-foramen magnum decompression is a relatively rare complication [18,19]. In such cases, prompt interventions, such as external drainage or shunting, are often recommended soon after the onset of symptoms [20,21]. In our patient, implementing a shunt successfully reduced the size of the epidural space and facilitated the recovery of brain volume.

In this case, the placement of a ventricular reservoir and closure of encephalocele during the neonatal period in a single procedure reduced ventricular size and increased brain volume without causing infection. However, there is a need for further research to determine the frequency of outpatient check-ups required for the patient afterward and the potential occurrence of complications other than external hydrocephalus. More importantly, future research is warranted to compare the prognosis of Chiari III patients undergoing reservoir placement to that of patients with shunting.

## Conclusions

This case of Chiari III malformation showcases significant hydrocephalus improvement following ventricular reservoir implantation in the newborn period. Subsequently, the emergence of secondary external hydrocephalus was effectively managed through subdural peritoneal shunting. The patient has thrived up to the age of five years, illustrating the potential and efficacy of this treatment approach; still, further research is needed to determine the optimal management strategy.
